# The Role of MASP1, MASP2, and Mannose-Binding Lectin in the Immune Response to Hepatitis B Vaccination in Infants

**DOI:** 10.3390/vaccines14010098

**Published:** 2026-01-20

**Authors:** Ayşe Esra Tapcı, İsmail Bulut, Serçin Taşar, Zeynep Kallimci, Kezban Çavdar Yetkin, Meliha Sevim, Oğuzhan Serin, Medine Ayşin Taşar, Mehmet Şenes, Bülent Alioğlu

**Affiliations:** 1 Department of Pediatrics, Ankara Training and Research Hospital, 06230 Ankara, Türkiye; sercin_gozkaya@yahoo.com (S.T.); zeynepdgrl@hotmail.com (Z.K.); melihakantekin@yahoo.com (M.S.); 2 Department of Neonatology, Etlik City Hospital, 06170 Ankara, Türkiye; isml7446@gmail.com; 3 Department of Biochemistry, Ankara Training and Research Hospital, 06230 Ankara, Türkiye; kezban.cavdar@hotmail.com (K.Ç.Y.); senesmehmet@yahoo.com (M.Ş.); 4 Department of Pediatric Emergency Medicine, Hacettepe University, 06230 Ankara, Türkiye; oguzhanserin@gmail.com; 5 Department of Pediatric Emergency Medicine, Ankara Training and Research Hospital, 06230 Ankara, Türkiye; aysintasar@yahoo.com; 6 Department of Pediatric Hematology and Oncology, Ankara Training and Research Hospital, 06230 Ankara, Türkiye; alioglub@gmail.com

**Keywords:** hepatitis B vaccine, mannose-binding lectin (MBL), mannose-associated serine protease 1 (MASP-1), innate immunity

## Abstract

**Background:** Hepatitis B vaccination is the most effective strategy for preventing chronic hepatitis B virus (HBV) infection; however, interindividual variability in vaccine-induced antibody responses remains a significant challenge in the field. Innate immune components, particularly lectin complement pathway proteins such as mannose-binding lectin (MBL), mannose-associated serine protease 1 (MASP-1), and mannose-associated serine protease 2 (MASP-2), may contribute to this variability in outcomes. This study aimed to evaluate the association between serum MBL, MASP-1, and MASP-2 levels, birth weight, and humoral response to hepatitis B vaccination in infants. **Methods:** This single-center prospective observational study included 37 term infants who received hepatitis B vaccinations at birth, 1 month, and 6 months of age according to the national immunization schedule. Venous blood samples were collected at month 6, before, and month 7 after the 3rd vaccine dose. Serum MBL, MASP-1, MASP-2, and antiHB levels were measured using commercial ELISA and chemiluminescence assays. Data were analyzed using non-parametric statistical tests and Spearman’s correlation analysis. **Results:** AntiHB levels increased significantly following vaccination (median Pre-3rdVac: 125.8 mIU/mL; Post-3rdVac: 609.7 mIU/mL; *p* < 0.001). MASP-1 concentrations also showed a significant Post-3rdVac increase (median Pre-3rdVac: 809.52 ng/mL; Post-3rdVac: 1133.93 ng/mL; *p* = 0.019). Birth weight was positively correlated with both MASP-1 levels (*r*_s_ = 0.492, *p* = 0.004) and changes in MASP-1 concentrations (*r*_s_ = 0.524, *p* = 0.002) after the third dose. In addition, MASP-1 levels were positively associated with antiHB levels (*r*_s_ = 0.385, *p* = 0.030) and Post-3rdVac antiHB titers (*r*_s_ = 0.493, *p* = 0.004). In contrast, serum MBL and MASP-2 concentrations were not significantly associated with antiHB levels before or after vaccination. **Conclusions:** MASP-1, but not MBL or MASP-2, is associated with the magnitude of the antibody response to hepatitis B vaccination in infants. These findings suggest that specific components of the lectin pathway may influence vaccine-induced immunity, independent of MBL. Further large-scale studies incorporating genetic and functional analyses are warranted to clarify the mechanisms by which lectin pathway proteins shape hepatitis B vaccine response.

## 1. Introduction

Hepatitis B is a viral infection that primarily affects the liver and can be transmitted through blood, body fluids, or vertical transmission from an infected mother to her infant. When chronicity develops, the infection may lead to severe complications such as cirrhosis and hepatocellular carcinoma [1]. According to the World Health Organization (WHO) Global Hepatitis Report, an estimated 254 million people lived with chronic hepatitis B infection in 2022, with approximately 1.2 million new cases reported annually. The same report indicates that hepatitis B is responsible for approximately 1.1 million deaths per year, most of which are attributable to cirrhosis and hepatocellular carcinoma [2]. Vaccination against hepatitis B, which can be administered at birth, remains the most effective strategy for preventing HBV infection and its long-term complications. However, a subset of individuals fails to mount an adequate antibody response despite receiving the recommended vaccine schedule.

Several factors have been implicated in reduced vaccine responsiveness, including advanced age, obesity, and a variety of primary immunodeficiency disorders, such as mannose-binding lectin (MBL) deficiency, leukocyte adhesion deficiency, chronic granulomatous disease, common variable immunodeficiency (CVID), hyper-IgM syndrome, and DiGeorge syndrome, as well as chronic conditions such as essential hypertension [3,4,5]. Notably, findings from twin studies suggest that 50–75% of the interindividual variability in vaccine-induced antibody responses is attributable to host genetic factors. Moreover, numerous immune-related genes, including those encoding complement cascade components, have been associated with differential responses to hepatitis B vaccination [6,7,8,9,10,11].

The complement system is an evolutionarily conserved, plasma-based defense mechanism that comprises three activation pathways. Mannose-binding lectin (MBL), a key pattern recognition molecule in the lectin pathway, binds to carbohydrate structures on microbial surfaces and initiates complement activation, thereby promoting opsonization and phagocytosis. Its functional partners, serine proteases mannose-associated serine protease 1 (MASP-1) and mannose-associated serine protease 2 (MASP-2), are associated with MBL/ficolin complexes and trigger downstream complement activation. Experimental models have demonstrated that MASP-1 plays a central role in activating MASP-2 and propagating lectin pathway signaling. This mechanism contributes to pathogen clearance and the modulation of adaptive immune responses [12,13].

MBL has been implicated in the pathogenesis and clinical course of both acute and chronic hepatitis B infection [14,15,16,17]. Furthermore, emerging evidence suggests that MBL may modulate humoral immune responses to certain vaccines, potentially through indirect effects on innate immune activation rather than direct antigen recognition [18,19,20,21]. Collectively, these findings indicate that the efficacy of HBV vaccination is shaped not only by adaptive immunity but also by innate immune system components.

However, human studies investigating the role of MBL, ficolin-2, and lectin pathway-associated proteases (such as MASP-1 and MASP-2) in HBV vaccine responsiveness remain limited, often involve adult populations, and yield inconsistent results. Therefore, particularly in pediatric cohorts, there is a clear need for larger, controlled, and multiparametric studies to elucidate the contribution of innate immune components to vaccine-induced immunity.

Although hepatitis B vaccination is highly effective worldwide, the immunological mechanisms underlying vaccine-induced protection during early infancy remain incompletely understood. During this developmental period, when adaptive immunity is still maturing, innate immune pathways may contribute disproportionately to shaping vaccine-induced immune responses. In this context, the aim of the present study was to investigate the relationship between hepatitis B vaccine responsiveness and innate immune factors by measuring serum levels of MBL, MASP-1, and MASP-2 and, where possible, evaluating relevant genetic polymorphisms. A deeper understanding of these interactions may help clarify the biological mechanisms underlying the responder and non-responder phenotypes following vaccination and provide a foundation for developing personalized immunization strategies in the future.

## 2. Materials and Methods

### 2.1. Study Design and Participants

This single-center, prospective observational study was conducted at our hospital between January 2023 and December 2023. Infants who received the monovalent hepatitis B vaccine according to the National Immunization Schedule of the Turkish Ministry of Health, specifically at birth (the birth dose of the hepatitis B vaccine was administered within the first 12 h after birth), 1 month, and 6 months of age were eligible for inclusion. The hepatitis B vaccine used in this study contained recombinant HBsAg produced in yeast, which lacks complex mammalian-type N-linked or O-linked glycosylation.

The sample size was determined by considering the number of participants commonly used in similar immunological response studies (30–60 subjects). Additional factors influencing sample size estimation included the low biological variability of MBL and MASP levels in infants, ethical limitations regarding blood sampling in this population, and potential challenges in longitudinal follow-up. Based on these considerations, a total of 37 infants were deemed sufficient to evaluate the expected effect size of the study.

### 2.2. Inclusion Criteria

- Term newborns with a gestational age of ≥37 weeks;
- Infants considered healthy at birth and throughout follow-up, with no major congenital anomalies or chronic diseases;
- Infants born to mothers who were HBsAg-negative (maternal screening included testing for HBsAg only; antiHBc and antiHBs were not routinely assessed);
- Infants not using any medications other than iron and vitamin D supplements during the study period;
- Infants without any acute infections during the follow-up;
- Written informed consent from parents or legal guardians.

### 2.3. Exclusion Criteria

- Preterm infants (<37 weeks of gestation);
- Infants born to mothers who were HBsAg-positive;
- Infants with suspected immunodeficiency;
- Infants receiving additional medications or those who did not complete the study.

### 2.4. Sample Collection and Processing

Venous blood samples were obtained from each infant at two time points.

1. At 6 months of age, immediately before administration of the third dose of the hepatitis B vaccine;
2. At 7 months of age, one month after the third dose of the hepatitis B vaccine.

Approximately 4 mL of venous blood was drawn at each time point from the antecubital region by a pediatric nurse using single-use evacuated blood collection tubes and transferred to plain polystyrene biochemistry tubes. The samples were transported to the laboratory within a maximum of 30 min and centrifuged at 4000 rpm for 5 min at room temperature.

The separated sera were transferred to sterile low-protein-binding polypropylene microtubes. A single freeze–thaw cycle was planned for all samples: sera were first stored at −20 °C for a short term and subsequently kept at −80 °C until the analysis.

Serum mannose-binding lectin (MBL), MASP-1, MASP-2, and antiHB levels were measured to assess the immune response to monovalent hepatitis B vaccination. MBL, MASP-1, and MASP-2 concentrations were determined using commercial ELISA kits according to the manufacturer’s instructions. The measurement ranges, lower limits of detection, and intra-/inter-assay coefficients of variation (%CV) were evaluated based on the quality control data provided by the manufacturers. AntiHB titers were examined using the enzyme-linked immunosorbent assay (ELISA) method (Abbott, Axsym, Germany), with values ≥10 mIU/mL considered protective.

All samples were analyzed in the same assay session under double-blind conditions.

The authors assert that all procedures contributing to this work comply with the ethical standards of the relevant national guidelines on human medical regulations and the Helsinki Declaration of 1975, as revised in 2008. The study was approved by the local ethics committee (approval number: E-22/1156 Ankara Training and Research Hospital Clinical Research Ethics Committee). Informed consent was obtained from all participants.

### 2.5. Statistical Analysis

Statistical analyses were performed using the Statistical Package for the Social Sciences (SPSS) software (version 26.0; SPSS Inc., Chicago, IL, USA). Descriptive variables are presented as counts, percentages, medians, interquartile ranges (IQRs), and minimum–maximum values. Normality of data distribution was assessed using the Kolmogorov–Smirnov and Shapiro–Wilk tests.

As the data were not normally distributed, the Mann–Whitney U test was used to compare independent groups, and the Wilcoxon signed-rank test was applied for paired comparisons. Statistical significance was set at *p* < 0.05.

Spearman’s rank correlation analysis was conducted to evaluate relationships between biomarkers and clinical–demographic variables. All correlation coefficients were presented in a correlation matrix, and a heatmap was generated to visually illustrate the observed associations. A significance level of *p* < 0.05 (two-tailed) was accepted for all analyses.

## 3. Results

The maternal, neonatal, and birth characteristics of the infants included in the study are shown in Table 1. The median age of the mothers was 27.5 years (interquartile range [IQR]: 24.0–30.5). The median birth weight of the newborns was 3290 g (IQR: 2987.5–3510), and the median gestational age was 38.5 weeks (IQR: 37.5–40.0). Of the participants, 68.8% were male, and 31.3% were female. The delivery mode was cesarean section in 59.4% of the cases and vaginal delivery in 40.6% of the cases.

**Figure 1:**
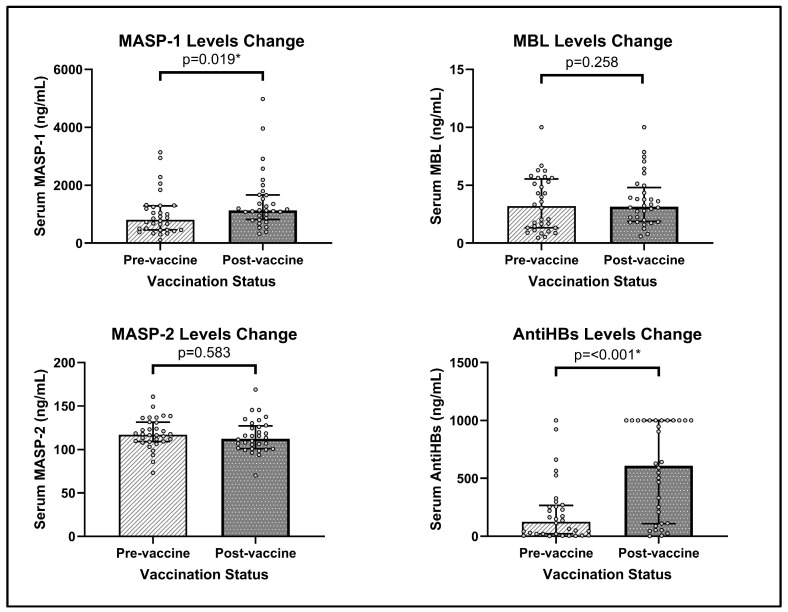
Serum concentrations of MBL, MASP-1, MASP-2, and antiHBs before (Pre-3rdVac) and after (Post-3rdVac) the third dose of hepatitis B vaccine. Bars represent the median values, and vertical error lines indicate the interquartile range (IQR). Each dot represents the serum measurement of each infant. * *p* < 0.05 was considered statistically significant.

Pre-3rdVac and Post-3rdVac levels of MBL, MASP-1, MASP-2, and antiHBs were evaluated at 6 and 7 months. No significant difference was observed in mannose-binding lectin (MBL) levels before and after vaccination (median Pre-3rdVac: 3.19 ng/mL, IQR: 1.33–5.47; Post-3rdVac: 3.15 ng/mL, IQR: 1.91–4.66; *p* = 0.258). Similarly, MASP-2 concentrations did not show a statistically significant change (Pre-3rdVac: 117 ng/mL, IQR: 109.11–131.33; Post-3rdVac: 112.42 ng/mL, IQR: 101.1–126.87; *p* = 0.583). In contrast, MASP-1 levels increased significantly following vaccination (Pre-3rdVac: 809.52 ng/mL, IQR: 458.33–1273.81; Post-3rdVac: 1133.93 ng/mL, IQR: 833.33–1663.05; *p* = 0.019). Additionally, hepatitis B surface antibody (antiHBs) levels rose markedly after vaccination (Pre-3rdVac: 125.8 mIU/mL, IQR: 20–262.5; Post-3rdVac: 609.7 mIU/mL, IQR: 110.48–1001; *p* < 0.001) (Table 2, Figure 1). Following the third dose of hepatitis B vaccination, 30 of 32 infants (93.8%) achieved antiHB levels ≥10 mIU/mL, while two infants (6.2%) remained below the seroprotective threshold (1.80 and 5.89 mIU/mL).

According to the Spearman correlation analysis, a significant positive correlation was observed between birth weight and gestational age among the 37 infants included in the study (*r*_s_ = 0.614, *p* < 0.001), indicating that a higher gestational age was associated with a greater birth weight. Furthermore, a significant positive correlation was found between Post-3rdVac (7th month) MASP-1 levels and birth weight (*r*_s_ = 0.492, *p* = 0.004), suggesting that infants with higher birth weights had higher MASP-1 levels after the final vaccine dose. The change in MASP-1 levels (ΔMASP-1) was also significantly correlated with birth weight (*r*_s_ = 0.524, *p* = 0.002), indicating that the increase in MASP-1 levels was greater in infants with higher birth weights.

**Figure 2:**
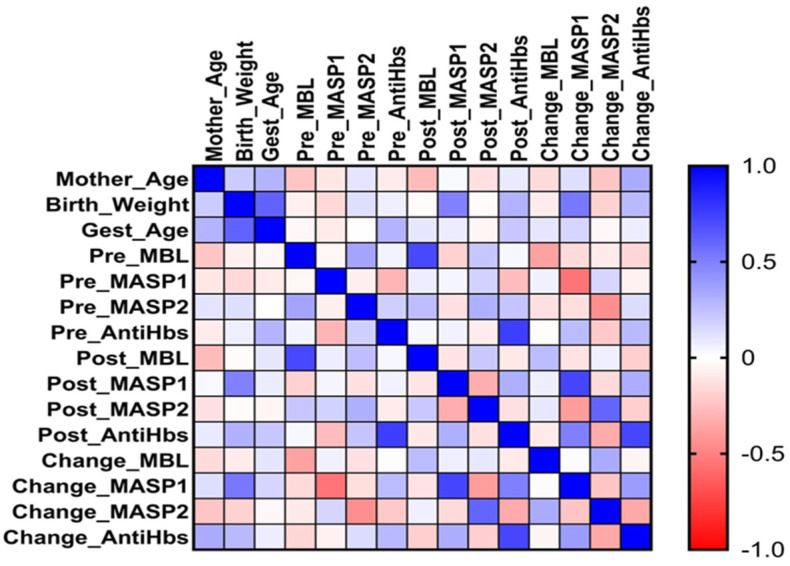
Pairwise Spearman correlation heatmap across continuous study variables.

As shown in Table 3 and Figure 2, a significant positive correlation was observed between the change in MASP-1 levels and the change in antiHB levels (*r*_s_ = 0.385, *p* = 0.030). This finding suggests that an increase in MASP-1 levels paralleled an increase in antiHB concentrations. Moreover, ΔMASP-1 was positively correlated with Post-3rdVac antiHB levels (*r*_s_ = 0.493, *p* = 0.004), indicating that infants who exhibited a greater increase in MASP-1 also had higher antiHB levels after vaccination.

In contrast, Pre-3rdVac MBL levels showed only a weak and statistically non-significant positive correlation with Pre-3rdVac antiHB levels (*r*_s_ = 0.04). Likewise, no meaningful correlation was found between MBL and Post-3rdVac antiHB levels (*r*_s_ = 0.03) or the change in antiHB levels (*r*_s_ = −0.16). These findings indicate that MBL concentrations are not associated with the antibody response to hepatitis B vaccination. The numerical correlation coefficients for all biomarkers are presented in Table 3, which details the relationships between all measured variables.

A visual representation of the Spearman correlation coefficients is shown in Figure 2. Positive correlations are shown in blue and negative correlations are shown in red. A strong positive association between birth weight and gestational age was observed. Birth weight was also positively correlated with Post-3rdVac MASP-1 levels and ΔMASP-1. Additionally, positive correlations between ΔMASP-1 and both ΔantiHB levels and Post-3rdVac antiHB concentrations were observed. This visualization supports the notion that birth weight, MASP-1 dynamics, and antibody responses may be interrelated indicators of the immune response to hepatitis B vaccination.

## 4. Discussion

In this study, we investigated the relationship between birth weight, considered a potential determinant of the immune response to monovalent hepatitis B vaccination, and the levels of MBL, MASP-1, MASP-2, and antiHBs. Our findings demonstrated that birth weight was significantly and positively associated with both Post-3rdVac MASP-1 levels and the magnitude of change in MASP-1 concentration. Furthermore, the increase in MASP-1 levels was positively correlated with both the change in antiHB titers and antiHB concentrations measured after the final vaccine dose. These results suggest that infants with a higher birth weight may develop a more robust immune response following hepatitis B vaccination.

Previous studies have demonstrated that low birth weight adversely affects the humoral immune response to hepatitis B vaccination. In particular, a study reported that following the administration of a combined vaccine series at 2, 4, and 6 months of age, very low-birth-weight (VLBW) infants exhibited lower seroprotection rates against hepatitis B after the booster dose compared with low-birth-weight (LBW) infants [22]. Similarly, another study showed that after completion of a three-dose hepatitis B vaccination schedule administered at 0, 1, and 6 months, LBW infants had significantly lower geometric mean antibody titers than those with normal birth weight [23].

In our study, no meaningful association was observed between MBL levels and the immune response to the vaccine. Neither Pre-3rdVac nor Post-3rdVac MBL concentrations correlated with antiHB titers. In contrast, infants who exhibited a greater increase in MASP-1 levels also demonstrated higher Post-3rdVac antiHB titers. This suggests that MASP-1 may be associated with the humoral response to hepatitis B vaccination in early infancy rather than directly shaping it.

Although the number of studies addressing this topic is limited, experimental data provide valuable insights into the immunomodulatory roles of MBL. Ruseva et al. (2009) [24] conducted a seminal animal study demonstrating that MBL deficiency influenced humoral immune responses in a manner highly dependent on genetic background. In mice lacking MBL-A and MBL-C, intravenous administration of soluble, glycosylated hepatitis B surface antigen (HBsAg) resulted in IgM antiHBsAg titers being threefold higher than those of control animals following primary immunization, and IgG titers were tenfold higher following booster immunization. Importantly, this study utilized glycosylated HBsAg in a murine model, which fundamentally differs from the recombinant, non-glycosylated HBsAg used in licensed hepatitis B vaccines administered to humans. Therefore, while the findings by Ruseva et al. highlight the potential immunomodulatory capacity of MBL under specific experimental conditions, direct extrapolation of these mechanisms to the present human vaccination study should be made with caution.

A more recent investigation focusing on SARS-CoV-2 variants demonstrated that MBL preserves its ability to recognize highly glycosylated viral spike proteins, including those of the Delta and Omicron variants. Interestingly, MBL binding significantly decreased after vaccination but increased markedly when serum IgG was removed [25]. These findings suggest that antibody levels can modulate MBL activity in the context of glycan-bearing viral antigens.

However, it should be noted that SARS-CoV-2 spike proteins are heavily glycosylated, whereas the hepatitis B vaccine used in the present study contains recombinant, non-glycosylated HBsAg. Therefore, while this study provides important conceptual insight into the interplay between antibodies and MBL, the underlying mechanisms cannot be directly extrapolated to the glycan-free vaccine protein used in our cohort.

Similarly, another experimental study evaluating the effect of MBL deficiency on antibody responses to conjugated and unconjugated group B Streptococcus (GBS) polysaccharide vaccines found that antigen-specific IgG responses were significantly higher in MBL-deficient mice immunized with a conjugated GBS PS–tetanus toxoid vaccine. These results indicate that MBL may exert a suppressive effect on T-cell–dependent antibody responses to conjugated vaccine antigens and that this inhibition is relieved in the absence of MBL [18]. Collectively, these findings highlight the complex role of MBL in adaptive immune responses across different vaccine platforms.

Contrary to these experimental observations, our study did not identify a significant association between baseline MBL levels and humoral responses to hepatitis B vaccination in infants. AntiHB titers were comparable between infants with higher and lower baseline MBL concentrations. These findings suggest that, within the context of early infancy, baseline MBL levels are not a strong determinant of the magnitude of the antibody response to hepatitis B vaccination.

Our findings are consistent with those of a large cohort study involving 568 participants (213 infants and 355 adolescents), which examined whether MBL2 gene polymorphisms and serum MBL levels influenced antibody responses following primary and booster acellular pertussis vaccinations. Variants in exon 1 of the MBL2 gene (codons 52, 54, and 57) and serum MBL concentrations were not associated with IgG responses to pertussis antigens (pertussis toxin, filamentous hemagglutinin, and pertactin). Even in adolescents with low MBL concentrations, the antibody responses were preserved. This study demonstrated that MBL deficiency or MBL2 polymorphisms did not alter antibody production or the persistence of humoral immunity after acellular pertussis vaccination [26]. Similar to our results, these findings suggest that humoral responses induced by protein-based vaccines may not be strongly dependent on MBL.

Although previous studies have emphasized a potential protective role of MBL in infections and its possible contribution to vaccine-induced immunity, our findings do not support a direct association between baseline MBL concentrations and the humoral response to hepatitis B vaccination in early infancy. In contrast, the significant associations observed between MASP-1 levels and both antiHB titers and ΔantiHB values suggest that MASP-1-related mechanisms may accompany vaccine-induced antibody responses independently of MBL. One possible explanation is that the third vaccine dose induces a robust antiHB response, leading to the formation of immune complexes with residual HBsAg, which may subsequently activate components of the complement system through IgG-mediated pathways, potentially contributing to increased MASP-1 activity. The absence of a similar association for MASP-2 suggests differential regulation within the lectin pathway. Nevertheless, this proposed mechanism remains speculative and warrants further investigation. Overall, these findings highlight a more nuanced role of lectin pathway components in early-life vaccine responses, emphasizing the importance of downstream effector molecules rather than upstream pattern-recognition factors alone.

A few limitations of this study should be acknowledged. The observational design does not allow causal inference, and systematic clinical follow-up after vaccination was not performed; therefore, potential associations between lectin pathway components and post-vaccination adverse events could not be assessed. In addition, the sample size was relatively modest, and functional assays were not included, which may limit the mechanistic interpretation of the findings.

## 5. Conclusions

This study explored the relationships between MBL, MASP-1, and MASP-2 and the humoral immune response to hepatitis B vaccination during early infancy. As expected, antiHB titers increased significantly following vaccination. While baseline MBL levels were not associated with vaccine responsiveness and MASP-2 showed no direct relationship with antiHB titers, changes in MASP-1 levels after the third vaccine dose were significantly associated with antibody responses. These findings suggest that components of the lectin pathway, particularly downstream effector molecules such as MASP-1, may accompany vaccine-induced immune activation even in the absence of a clear contribution from MBL. However, the observational nature of this study precludes causal inference, and the mechanisms underlying MASP-1 elevation remain to be fully elucidated. Further studies incorporating larger cohorts, genetic analyses, and functional assays are warranted to clarify the distinct roles of MBL, MASP-1, MASP-2, and other lectin pathway proteins in modulating vaccine responses in early life.

## Figures and Tables

**Table 1:** Maternal, neonatal, and delivery characteristics.

Mother’s Age, years [Median (Q1, Q3)]	27.5 (24.0–30.5)
Birth Weight, g [Median (Q1, Q3)]	3290 (2987.5–3510)
Gestational Age, weeks [Median (Q1, Q3)]	38.5 (37.5–40.0)
Gender, Female [*n* (%)]	10 (31.3%)
Gender, Male [*n* (%)]	22 (68.8%)
Delivery Mode, C/S [*n* (%)]	19 (59.4%)
Delivery Mode, Vaginal [*n* (%)]	13 (40.6%)

**Table 2:** Median concentrations of MBL, MASP-1, MASP-2, and antiHB levels before and after hepatitis B vaccination.

	Pre-3rdVac	Post-3rdVac	*p*-Value
MBL (ng/mL)	3.19 (1.33–5.47)	3.15 (1.91–4.66)	0.258
MASP1 (ng/mL)	809.52 (458.33–1273.81)	1133.93 (833.33–1663.05)	0.019 *
MASP2 (ng/mL)	117 (109.11–131.33)	112.42 (101.1–126.87)	0.583
AntiHBs (mIU/mL)	125.8 (20–262.5)	609.7 (110.48–1001)	<0.001 *

Values represent medians (Q1–Q3). The Wilcoxon signed-rank test was used for statistical comparisons. MBL: mannose-binding lectin; MASP1: mannan-binding lectin serine protease-1; MASP2: mannan-binding lectin serine protease-2; Anti-HBs: hepatitis B surface antibody. * *p* < 0.05 was considered statistically significant.

**Table 3:** Spearman’s rank correlation coefficients among study variables.

	Mother _Age	Birth_Wei ght	Gest_A ge	Pre_M BL	Pre_MA SP1	Pre_MA SP2	Pre_Anti Hbs	Post_MBL	Post_MA SP1	Post_MA SP2	Post_Anti Hbs	Change_MBL	Change_M ASP1	Change_M ASP2	Change_Ant iHbs
Mother_Age	1.00	0.20	0.30	−0.23	−0.10	0.10	−0.08	−0.27	0.03	−0.13	0.08	−0.14	0.13	−0.23	0.33
Birth_Weight	0.20	1.00	0.61	−0.06	−0.15	0.13	0.06	−0.02	0.49	−0.02	0.30	−0.08	0.53	−0.18	0.27
Gest_Age	0.30	0.61	1.00	−0.03	−0.08	0.00	0.30	0.10	0.07	−0.04	0.22	0.10	0.16	−0.04	0.07
Pre_MBL	−0.23	−0.06	−0.03	1.00	−0.04	0.36	0.04	0.72	−0.18	0.22	0.03	−0.37	−0.15	−0.09	−0.16
Pre_MASP1	−0.10	−0.15	−0.08	−0.04	1.00	−0.07	−0.29	0.07	0.04	0.18	−0.27	0.06	−0.54	0.16	−0.06
Pre_MASP2	0.10	0.13	0.00	0.36	−0.07	1.00	0.19	0.26	−0.12	0.31	0.23	−0.13	−0.13	−0.45	0.13
Pre_AntiHbs	−0.08	0.06	0.30	0.04	−0.29	0.19	1.00	0.03	0.05	−0.08	0.76	−0.01	0.27	−0.21	0.27
Post_MBL	−0.27	−0.02	0.10	0.72	0.07	0.26	0.03	1.00	−0.11	0.21	−0.09	0.26	−0.11	0.06	−0.19
Post_MASP1	0.03	0.49	0.07	−0.18	0.04	−0.12	0.05	−0.11	1.00	−0.33	0.31	0.06	0.73	−0.15	0.32
Post_MASP2	−0.13	−0.02	−0.04	0.22	0.18	0.31	−0.08	0.21	−0.33	1.00	−0.12	0.09	−0.39	0.61	−0.19
Post_AntiHbs	0.08	0.30	0.22	0.03	−0.27	0.23	0.76	−0.09	0.31	−0.12	1.00	−0.09	0.49	−0.33	0.73

## Data Availability

The data generated in this study are fully presented within the article. Due to privacy/ethical considerations, no additional data are available beyond what is included in the manuscript.
